# Influence of Load in Formaldehyde and Acetaldehyde Emissions and Performance of a Diesel Engine Using Various Diesel–Vegetable Oil and Diesel–Biodiesel Blends

**DOI:** 10.1021/acsomega.5c04275

**Published:** 2025-09-01

**Authors:** Oscar Edwin Piamba Tulcán, Roberto Guimarães Pereira, Carlos E. Fellows

**Affiliations:** † Universidad Nacional de Colombia, University City, Carrera 30 # 45-03, 111321 Bogotá, Colombia; ‡ Fluminense Federal University - TEM/PGMEC/PGEB, Rua Passo da Pátria 156, CEP 24210-240 Niterói, RJ, Brazil; § Departmento de Física, Instituto de Ciências Exatas - ICEx, 28110 Universidade Federal Fluminense, Campus do Aterrado, Volta Redonda, Niterói, RJ 27213-45, Brazil

## Abstract

The objective of this study is to investigate the impact of load on formaldehyde and acetaldehyde emissions, as well as the performance of a diesel engine when utilizing a variety of diesel blends with vegetable oils and biodiesels. The oils and biodiesels under examination were derived from canola, soy, palm, coconut, and beef tallow. The analysis of the aldehyde emissions was conducted by using the differential optical absorption spectroscopy (DOAS) method. The biodiesels were blended with diesel in specific proportions, and the emissions of formaldehyde and acetaldehyde were compared. The findings indicated that the incorporation of triglycerides and biodiesel into diesel fuel led to an observed increase in formaldehyde and acetaldehyde emissions. Furthermore, the types of triglycerides and biodiesel employed exerted an influence on formaldehyde and acetaldehyde emissions as the engine load increased. In general, formaldehyde and acetaldehyde emissions were higher at higher loads than at lower loads. Additionally, formaldehyde emissions exceeded those of acetaldehyde emissions. Furthermore, the specific fuel consumption for the fuels under investigation decreased with increasing load.

## Graphical abstract

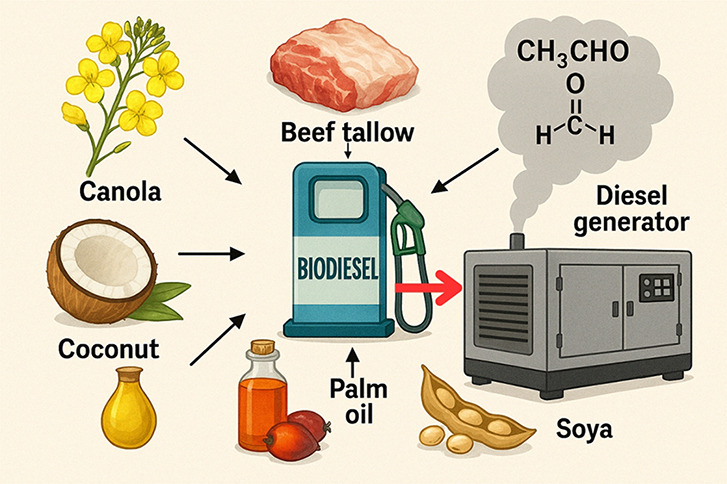

## Introduction

The interest in the utilization of biodiesels derived from vegetable oils or animal fats as an alternative fuel source for petroleum-based diesel engines has increased due to the fact that biodiesels possess properties that are analogous to those of diesel engines, in addition to exhibiting characteristics of renewability, biodegradability, and a reduction in the emission of certain pollutants, such as SO_2_. In general, the emissions of regulated pollutants in the exhaust are widely studied, and the results indicate that biodiesels perform better than conventional diesel in terms of CO, HC, and particulate emissions. However, there are limited and inconsistent data available for unregulated pollutants, such as carbonyl compounds, which are also important indicators for evaluating the fuels available for vehicles. In order to gain a deeper understanding of biodiesel, this study compares the effects of biodiesel blends on the chemical aldehyde emissions of diesel engine exhausts with those of diesel fuel. Carbonyl compounds, in particular formaldehyde and acetaldehyde, are released directly into the atmosphere by a multitude of sources.^1^ These two compounds are particularly prevalent among atmospheric carbonyl compounds and exert a significant influence on the atmospheric chemistry of polluted areas.^2^ The combustion of biofuels is a primary source of both formaldehyde and acetaldehyde, which are released directly and indirectly into the air. These compounds are present in trace amounts in virtually all combustion processes, making them highly susceptible to atmospheric release. With the increasing use of alternative fuels derived from biomass, such as biodiesel, there has been a corresponding increase in interest among researchers in studying the vehicle emissions of these compounds.^3^ Biodiesel is a renewable biofuel derived from biomass. It is designed for use in internal combustion engines with compression ignition and offers a potential replacement for fossil fuels, either partially or entirely. Although numerous studies, including several of our own,^4–8^ on diesel–biodiesel blends used in diesel engines have concentrated on regulated emissions such as hydrocarbons (HC), carbon monoxide (CO), and nitrogen oxides (NO_*x*_), there is a noticeable absence of research on unregulated emissions. While numerous studies have investigated biofuel emissions, a clear consensus on the influence of engine load on formaldehyde and aldehyde formation remains elusive, with contradictory findings reported across the literature when utilizing blends of diesel with coconut oil, palm oil, beef tallow, and coconut biodiesel. In this context, the present study examines the influence of engine load on formaldehyde and acetaldehyde emissions as well as the performance of a diesel engine utilizing blends of diesel–vegetable oil, diesel–animal fat, and diesel–biodiesel derived from various raw materials (including soybean, coconut, palm, canola, and beef tallow). Formaldehyde and acetaldehyde concentrations were measured by using differential optical absorption spectroscopy (DOAS).

## Materials and Methods

The experimental methodology comprised three stages: (1) selection and characterization of raw materials, biofuel processing, and fuel mixtures; (2) evaluation of engine performance using an electrical load test bench; and (3) quantification of acetaldehyde and formaldehyde exhaust emissions.

### Fuel Choice and Characterization

Five raw materials—beef tallow, palm oil, coconut oil, soybean oil, and canola oil—were selected for this work based on their chemical composition and global market availability for biomass energy transformation and biodiesel production. Beef tallow is characterized by a high proportion of saturated, long-chain fatty acids, while coconut oil is rich in short-chain saturated fatty acids. Palm oil contains a balanced mix of saturated and unsaturated long-chain fatty acids, whereas soybean and canola oils are predominantly composed of unsaturated fatty acids. This compositional variation directly influences the physicochemical properties of both the triglycerides and the resulting biodiesel.^9^

Palm oil, a highly productive oilseed with a well-established global market, has demonstrated favorable results in the production of both methyl and ethyl biodiesel. The production of this oilseed is becoming increasingly prevalent in Brazil, with extensive production areas located in the Northeast and North and Northeast regions.

Coconut palms, another highly productive variety, are extensively cultivated in Brazil, particularly in the Northeast, with numerous large-scale plantations in operation. Soybean, the most widely cultivated oilseed in Brazil, is characterized by its unsaturated composition. Currently, soybean-based biodiesel production is the most prevalent, with the majority of production facilities located in the southeastern, central, and southern regions. However, the price of this oil is increasing, which may result in its loss of competitiveness in the market for raw materials used in biodiesel production.

Canola oil is produced in Brazil for culinary purposes. Canola is often considered a substitute for soy, especially in the southern and central regions of the country. Canola biodiesel is currently the dominant type in Europe’s biodiesel market. This presents an opportunity for Brazilian products to gain access to foreign markets. Beef tallow is currently employed as a crucial raw material in the production of biodiesel in Brazil. However, its elevated cloud point is not a significant concern in tropical weather regions. The price per ton of animal fat is comparatively lower than that of soybean oil, making it a viable option for biodiesel production in the southern region.

For this study, refined soybean oil and canola oil (food-grade) were procured from local commercial suppliers, while unrefined palm and coconut oils were sourced directly from regional producers. Beef tallow, obtained as a byproduct from a local butcher, was processed under controlled conditions to ensure consistency. Conventional diesel fuel was provided by the Distributed Energy Generation Laboratory at the Fluminense Federal University. All feedstock processing and fuel synthesis—including diesel–triglyceride blends (vegetable oils/tallow with diesel) and diesel–biodiesel blends—were conducted at the Thermo-Sciences Laboratory of the Mechanical Engineering Department at Fluminense Federal University.

Existing literature strongly advises against exceeding 30% vegetable oil in diesel blends due to risks of engine deposits, injector coking, and lubricant contamination.^10^ While biodiesel (B100) is recognized as a full diesel substitute.^11^ Unprocessed vegetable oils exhibit elevated viscosity and density, which impair atomization and combustion efficiency.^12,13^ To mitigate these risks, our study adopted a conservative threshold of 15% vegetable oil or tallow by volume in diesel blends, designated as “O15” (e.g., O15 refers to 15% oil/tallow +85% diesel). Similarly, “BXX” denotes biodiesel–diesel blends where “XX” represents the biodiesel volume percentage. Tested blends included B15 (15% biodiesel), B100 (pure biodiesel), and B0 (pure diesel) as baseline references.

This methodology aligns with recent findings that emphasize viscosity reduction through blending^14^ while addressing long-term engine durability concerns associated with unmodified vegetable oils.^12^

Following feedstock acquisition, unrefined palm and coconut oils were pretreated via microfiltration at 40 °C to remove particulate matter. Beef tallow underwent a two-stage process: initial heating to 80 °C to eliminate moisture and facilitate triglyceride separation, followed by microfiltration at 60 °C to further purify the rendered tallow. All processed oils, including the refined soybean and canola oils, were then blended with diesel fuel at a controlled temperature of 35 °C in a sealed vessel to ensure homogeneity.

Concurrently, a portion of the processed feedstocks was dedicated to biodiesel production. Anhydrous methyl alcohol was employed in transesterification reactions to convert the oils into fatty acid methyl esters (FAME), or biodiesel. To accommodate varying feedstock characteristics, the oils were divided into two groups: (1) refined soybean and canola oils, which underwent standard base-catalyzed transesterification; and (2) unrefined palm and coconut oils, which required a two-step process involving initial acid-catalyzed esterification (using sulfuric acid to prevent free fatty acid saponification), followed by base-catalyzed transesterification. This dual-pathway approach optimized biodiesel conversion efficiency and product quality across a diverse range of feedstocks.

### Engine Test Bench

The engine test bench was designed to conduct comprehensive performance evaluations and emission measurements. It comprises a diesel engine-generator setup, a variable resistive load system, and power measurement equipment. An optical spectrometer was integrated into the system to detect and quantify pollutants using the differential optical absorption spectroscopy (DOAS) method, which is detailed in the subsequent subsection.

The diesel engine-generator utilized in this study is a Branco BD 2500 model, capable of generating a nominal power output of 2 kW. This setup includes a Branco BD 5.0 engine coupled with an electrical generator and a control panel that allows voltage selection between 115 and 230 V. The generator features an electronic control system that regulates engine speed and provides thermal protection, ensuring stable operation across varying loads. Under full throttle conditions, the voltage output remains consistent with any deviations adjustable via the injection pump governor lever. The specifications of the generator and diesel engine are summarized in Tables 1 and 2.

**Table 1:** Characteristics of the Branco BD 2500 Generator

Branco BD 2500 generator	technical specification
maximum power	2.5 KVA
rated power	2.2 KVA
output voltage	110/220 V
weight	53 kg
phases	single-phase

**Table 2:** Characteristics of the Branco BD 5.0 Engine

Branco BD 5.0 engine	technical specification
type	single cylinder, 4 stroke, direct fuel injection
maximum power	5.0 CV at 3600 rpm
rated power	4.2 CV at 3600 rpm
rated rotation	3000 rpm
displacement	210 cc
fuel tank capacity	2.5 L
weight	27 kg

To facilitate precise fuel consumption measurements, the fuel tank was modified to enable gravimetric fuel consumption monitoring using a Nucleo Analytical Balance, 520 g, 0.01 g. The electrical load was applied through a resistance load bank consisting of 100 and 150 W resistances, allowing for four distinct load levels: 20, 35, 50, and 65% of the maximum nominal load, corresponding to 400, 700, 1000, and 1300 W. Test conditions are presented in Table 3.

**Table 3:** Engine Test Conditions: Fuels and Load Level

feedstock	diesel–oil blend	diesel–biodiesel blend		load level			
canola	O15	B15	B100	20%	35%	50%	65%
coconut	O15	B15	B100	20%	35%	50%	65%
palm	O15	B15	B100	20%	35%	50%	65%
soybean	O15	B15	B100	20%	35%	50%	65%
beef tallow	O15	B15	B100	20%	35%	50%	65%
diesel				20%	35%	50%	65%

Instantaneous power, current frequency, voltage, and electric current were measured using a CCK 4300 device. This meter features a voltage measurement range from 30 to 500 V AC, a current measurement range from 20 mA to 5 A, an accuracy class of 0.5%, and communication via an RS-485 parallel port, with an operating temperature range from 0 to 50 °C.

### Exhaust Emission Measurements: The DOAS Method

Differential optical absorption spectroscopy (DOAS) is a spectroscopic technique used to determine concentrations of trace gases by measuring their narrow, specific absorption bands in the UV and visible spectra.^15^ In this methodology, the experimental setup is similar to that of classical absorption spectroscopy, but the data analysis approach differs. This method can be active or passive. In active DOAS, the researcher controls the light source used and the path length, whereas in passive DOAS, such as when utilizing sunlight, the light source is beyond the researcher’s control, as also the path length. In this study, active DOAS has been employed.

The DOAS methodology was developed primarily for analyzing atmospheric composition, where various scattering agents complicate the direct application of the Beer–Lambert equation.

$$I=I_{0}\exp\left(-\sum_{i}\int \rho_{i}\beta_{i}ds\right)$$ 1

where *I*_0_ is the intensity of the radiation source, ρ is the density of the gas, β is the absorption cross section, and *s* is the path traveled by the radiation. The subscript *i* denotes the different gas species, assuming that the medium consists of several species. Bearing in mind that in our case the path traveled is well known and constant and that the active DOAS method is used to measure column density, the integral gives a single parameter called column density σ:

$$\sigma_{i}=\int \rho_{i}ds$$ 2

The equation then becomes

$$I=I_{0}\exp\left(-\sum_{i}\beta_{i}\sigma_{i}\right)$$ 3

The quantity β_*i*_σ_*i*_ is called the optical depth δ and can be calculated using the expression:

$$\delta =\ln\left(\frac{I_{0}}{I}\right)=\sum_{i}\beta_{i}\sigma_{i}$$ 4

taking into account that the intensity *I*_0_ is known, since we are using active DOAS and the light source is always the same, and its spectrum can be determined in advance.

This is where the DOAS method differs from traditional absorption methods. All the absorption cross sections, as well as the spectrum of the lamp used in active DOAS, have a “fast” part and a “slow” part, so eq 3 can be rewritten as

$$I=I_{0}\exp\left(-\sum_{i}(\beta_{is}+\beta_{if})\sigma_{i}\right)$$ 5

where β_*i*s_ is the “slow” part of the absorption cross section and β_*i*f_ is the “fast” part of the absorption cross section. The slow part is retained from the spectrum, and the one that remains is the fast one, which is called the differential cross-section. Thus:

$$\delta_{f}+\delta_{s}=\ln{\left(\frac{I_{0}}{I}\right)}_{f}+\ln{\left(\frac{I_{0}}{I}\right)}_{s}=\sum_{i}\beta_{if}\sigma +\sum_{i}\beta_{is}\sigma$$ 6

considering that the column density σ_*i*_ is the same for all gases and equal to σ.

Removing the slow components by simply fitting a polynomial to the spectrum and then subtracting it, and considering the wavelength dependence, produces a matrix equation with which the inversion can be made:

$$\delta_{f}(\lambda )=\sum_{i}\beta_{if}\sigma$$ 7

With the knowledge of the cross sections of the gaseous species of interest and calculating their differential cross sections, it is possible to obtain their concentrations through a straightforward matrix inversion. An example of the cross-section and its differential cross-section is illustrated in Figure 1 for the case of the CH_2_O molecule.

**Figure 1:**
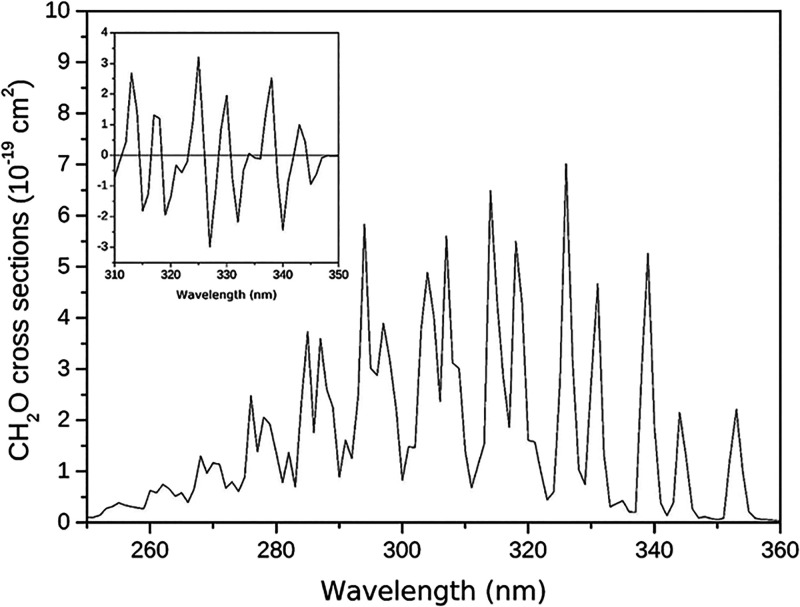
Absorption cross-section of the CH_2_O molecule. In the inset, one can see the differential cross-section for the region between 310 and 350 nm.

One wonders what the advantage is of using the DOAS method instead of traditional absorption spectroscopy. The answer is simple. We have to consider that during the measurement process, there is a turbulent flow of gases emitted by the generator set, where we also have to consider the large amount of particulates that will be emitted when the fuel is burnt. Both turbulence and the presence of particulates, which produce Mie and Rayleigh scattering, have a certain influence on absorbance as a function of wavelength. However, these influences do not produce abrupt variations in absorbance as a function of wavelength (Mie scattering, for example, varies with λ^–α^, where α = 0.5–2.5). In this way, the active DOAS method used in our study proved to be perfectly adapted to the conditions under which the pollutant gas species were measured. The experimental setup used in the DOAS system in this work consists of the following components:

- Newport 67005 Arc Lamp Housing with a xenon arc lamp, of 500 W, with UV–visible spectrum ranging from 200 to 500 nm;
- Container for specimen collection: aluminum tube with quartz windows;
- Quartz lens with a focal length of 38 mm;
- Miniature Spectrometer Ocean Optics USB 4000, covering the spectral region between 200 and 950 nm;
- Quartz optic fiber, length of 2.0 m, and 600 μm core diameter;
- Acquisition Software of the Spectrum Ocean Optics, SpectraSuite.

Figure 2 shows the experimental apparatus used in the engine tests and exhaust emissions measurements. More details on the DOAS method can be found in the study of Platt et al.,^15^ and the experimental apparatus used in this work can be found in Tulcan.^16^

**Figure 2:**
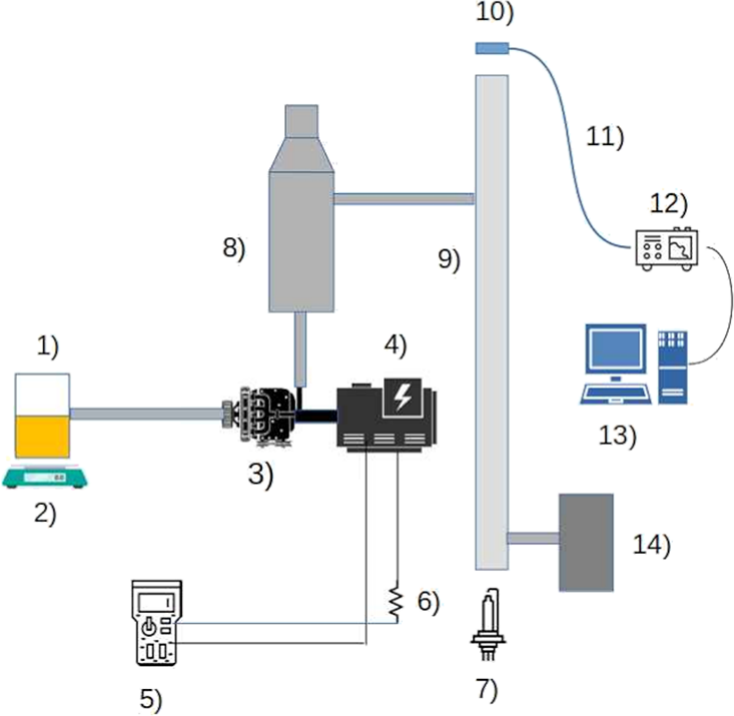
Experimental apparatus used in the engine tests and the emission measurements: (1) Fuel, (2) analytical balance, (3) engine, (4) electric generator, (5) multimeter, (6) resistive charge, (7) xenon lamp, (8) chimney, (9) exhaust gas sample device, (10) optics, (11) optical fiber, (12) spectrometer, (13) computer, and (14) gas analyzer.

## Results

### Properties of the Vegetable Oils and Animal Fat

The physicochemical properties of the vegetable oils (canola, coconut, palm, and soybean) and beef tallow evaluated in this study were measured at the Thermosciences Laboratory and the Rheology Laboratory of the Federal Fluminense University (Brazil). The calorific value determinations were carried out at the Fuels Laboratory of the National University of Colombia. Following the standardized methodologies described in Table 4, the properties of conventional diesel, vegetable oils, and beef tallow were obtained, as summarized in Table 5.

**Table 4:** Technical Standards Associated with the Characterization Tests

property	technical standard
density	ASTM D 4052 Density and Relative Density of Liquids by Digital Density Meter
viscosity	ASTM D 445 Standard Test Method for Viscosity of Transparent and Opaque Liquids
cloud point	ASTM D2500 Test Method for Cloud Point of Petroleum Products
pour point	ASTM D97 Test Method for Pour Point of Petroleum Products
flash point	ASTM D 93 Standard Test Methods for Flash Point by Pensky-Martens Closed Cup Tester
heat of combustion	ASTM 240 Test Method for Heat of Combustion of Petroleum Products
copper strip corrosion	ASTM D130 Test Method for Copper strip corrosion of Petroleum Products

**Table 5:** Properties of Diesel, Vegetable Oils, and Animal Fat^a^,^b^

property	diesel	canola oil	coconut oil	palm oil	soybean oil	beef tallow
density, kg/L (20 °C)	0.8570	0.9172	0.9139	0.9151	0.9237	0.9098
viscosity, mm^2^/s (40 °C)	4.689	34.930	31.846	66.260	31.410	n.d.
cloud point, °C	2	–1	25	24	–2	43
pour point, °C	–12	–18	15	1	–20	22
flash point, °C	82	n.d.	n.d.	n.d.	332	n.d
higher heating value, kJ/kg	42,800	40,640	38,430	39,110	39,417	39,250
copper strip corrosion	1a	1a	1a	1a	1b	1b
molecular mass, g/mol	170	880.8	714.5	850.5	875.1	861.6

a The expanded uncertainty of measurements are: density = ±0.00008 kg L^–1^; viscosity = ±0.006 mm^2^ s^–1^; flash point = ±2.1 °C; cloud point = ±1.5 °C and pour point = ±1.8 °C.^17,18^

b n.d. = not determined.

All reported values represent the mean of at least three independent experimental trials. The measured physicochemical properties of diesel and triglycerides align with typical ranges documented by Knothe et al.,^10^ closely matching established literature averages.

### Properties of the Biodiesels

The biodiesel samples were characterized collaboratively by the Federal Fluminense University and the National University of Colombia. Table 6 presents the evaluated physicochemical properties of the biodiesel fuels, while Table 7 details their fatty acid methyl ester (FAME) profiles.

**Table 6:** Properties of Diesel and Biodiesels^a^

property	diesel	canola biodiesel	coconut biodiesel	palm biodiesel	soybean biodiesel	beef tallow biodiesel
density, kg/L (20 °C)	0.8570	0.8839	0.8773	0.8774	0.8903	0.8647
viscosity, mm^2^/s (40 °C)	4.689	4.538	3.421	4.872	4.233	5.027
cloud point, °C	2	1	3	10	0	15
pour point, °C	–12	–11	–6	0	–6	11
flash point, °C	82	156	156	163	150	158
higher heating value, kJ/kg	42,800	42,110	39,840	41,700	41,685	42,365
copper strip corrosion	1a	1a	1a	1a	1a	1a
molecular mass, g/mol	170	295.1	239.5	284.8	293.1	302.5

a The expanded uncertainty of measurements are: density = ±0.00008 kg L^–1^; viscosity = ±0.006 mm^2^ s^–1^; flash point = ±2.1 °C; cloud point = ±1.5 °C and pour point = ±1.8 °C.^17,18^

**Table 7:** Composition in the Percentage of Fatty Acids of Biodiesels

fatty acid	canola	coconut	palm	soybean	beef tallow
lauric (C12:0)	0.0	45.3	0.0	0.0	0.0
myristic (C14:0)	0.0	32.5	1.9	0.0	4.0
palmitic (C16:0)	6.3	11.9	44.2	11.6	27.4
palmitoleic (C16:1)	0.6	0.0	0.0	0.1	0.0
steriac (C18:0)	2.7	1.7	4.5	3.2	25.6
oleic (C18:1)	60.4	3.3	39.6	20.4	39.1
linoleic (C18:2)	21.3	5.3	9.8	59.7	1.6
linolenic (C18:3)	8.7	0.0	0.0	5.0	2.2
iodine value^a^	112	12	51	134	42

a Iodine value was calculated by means the AOCS Official Method Ce 1c-85 “Calculated Iodine Value”.^19^

The properties of conventional diesel and biodiesel derived from canola, coconut, palm, soybean, and beef tallow were determined using the standardized methods, as referenced in Table 4. The obtained physicochemical parameters for diesel and biodiesel correlate well with benchmark values reported by Knothe et al.,^10^ exhibiting minimal deviation from literature-derived averages. Furthermore, the observed fatty acid concentration profiles correspond to characteristic compositions for these feedstocks, as documented in prior studies.

### Performance and Pollutant Emission Tests

#### Specific Fuel Consumption, SFC

Figure 3 illustrates the specific fuel consumption (SFC) behavior for diesel–triglyceride mixtures of O15 at various loads. The SFC patterns demonstrate similar trends for both pure diesel and O15 diesel–triglyceride mixtures.

**Figure 3:**
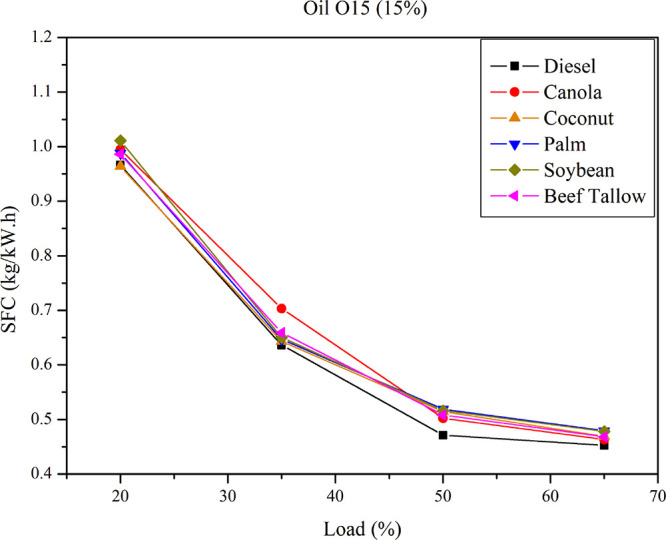
SFC for diesel–triglyceride mixtures of O15 at different loads.

Figure 4 presents the SFC for B15 diesel–biodiesel mixtures across different loads. The data indicate comparable SFC behavior between pure diesel and B15 diesel–biodiesel mixtures. At higher loads, the SFC values converge for all biodiesel blends (except for the coconut biodiesel), exhibiting slightly elevated values compared to those of pure diesel.

**Figure 4:**
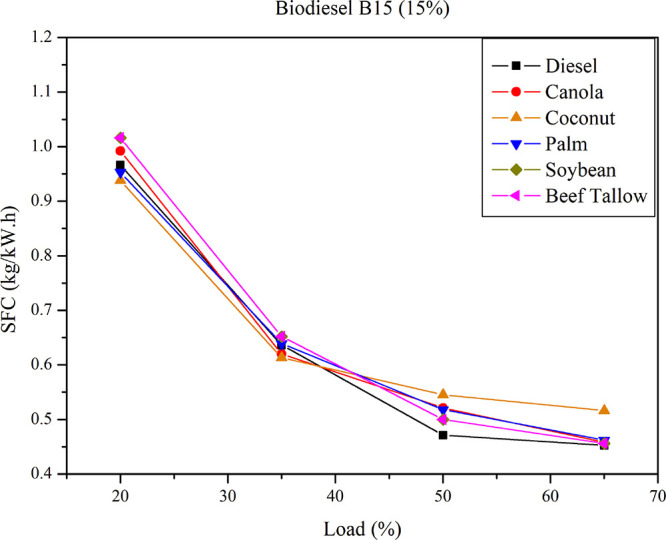
SFC for B15 diesel–biodiesel mixtures at different loads.

Figure 5 depicts the SFC comparison between B100 (pure biodiesel) and pure diesel at varying loads. For all tested biodiesels, SFC values exceeded those of pure diesel across all load conditions. Notably, coconut and soybean biodiesels demonstrated an approximately 20% higher SFC at 20% load, increasing to approximately 30% higher at 50% load relative to pure diesel.

**Figure 5:**
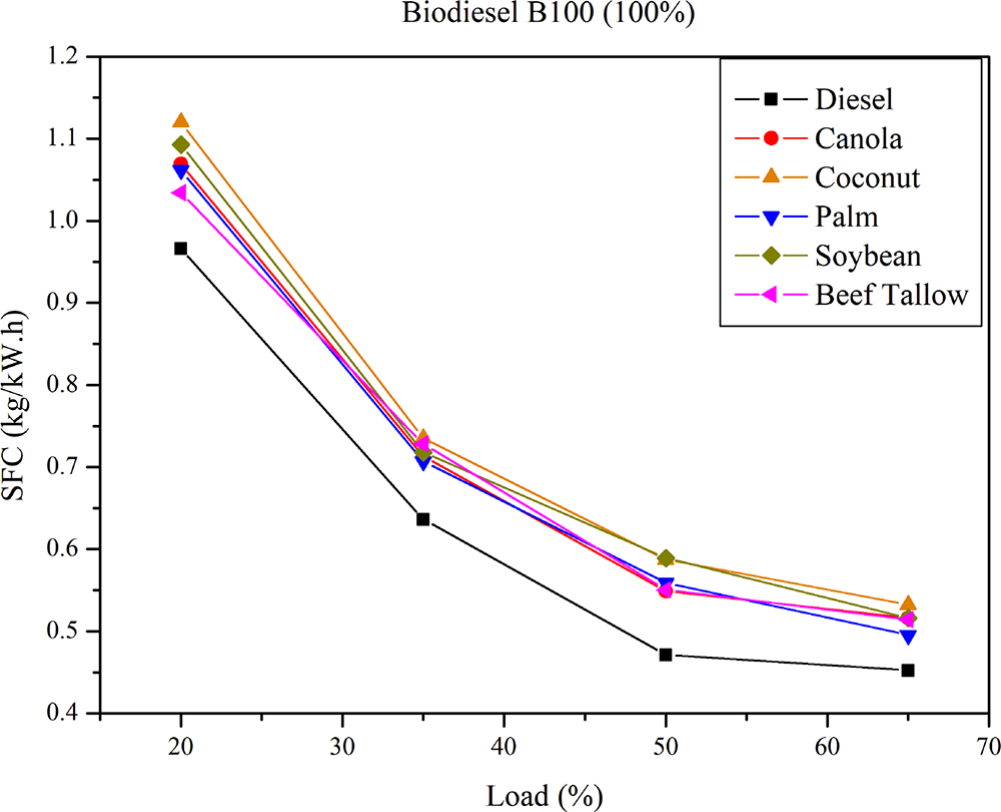
SFC for diesel and pure biodiesel (B100) at different loads.

The specific fuel consumption (SFC) exhibited consistent patterns across all examined blends, including both diesel–triglyceride and diesel–biodiesel mixtures. In all tests, a decrease in SFC was observed with increasing engine load, a finding consistent with previously reported data for engines operating on diesel, biodiesel, and various diesel–biodiesel and diesel–vegetable oil blends.^5,20–22^

A comparative analysis of engine performance, based on the Specific Fuel Consumption (SFC) of a 15% diesel–oil blend and a diesel–biodiesel blend, indicates an increase in SFC for both blends relative to pure diesel. When comparing SFC values between oil blends and biodiesel blends derived from the same feedstock, it is observed that SFC is slightly higher for oil blends than for biodiesel blends, with the difference reaching up to 8%. On average, oil blends result in 1.2% higher SFC than biodiesel blends. The influence of feedstock on SFC between diesel/oil and diesel/biodiesel blends is also apparent. Blends derived from beef tallow and palm oil exhibited the largest differences in SFC (an average of 4%), whereas blends from soybeans and canola displayed minimal differences (approximately 1% on average). This behavior can be attributed to several factors, notably the higher viscosity and lower calorific value of oils—characteristics that are improved via the transesterification process for biodiesel production, thereby enhancing engine combustion processes.^23,24^

An analysis of the impact of biodiesel feedstock on SFC reveals that coconut-based biodiesel exhibits the highest SFC values, while beef-tallow-based biodiesel demonstrates the lowest. This trend suggests that the molecular size of the biofuel influences SFC: fuels with lower molecular masses tend to have higher SFC values, whereas those with higher molecular masses exhibit lower SFC values. As observed in the fuels studied, the molecular mass is proportional to the higher heating value (HHV)—lower molecular mass corresponds to lower calorific value, demanding greater fuel consumption to generate equivalent work, as reported by authors such as Lee and Cho.^25^

#### Formaldehyde Emission

Figure 6 presents formaldehyde emissions for diesel–triglyceride mixtures of the O15 class under varying load conditions. The data show that formaldehyde emissions from O15 mixtures exceed those of pure diesel fuel.

**Figure 6:**
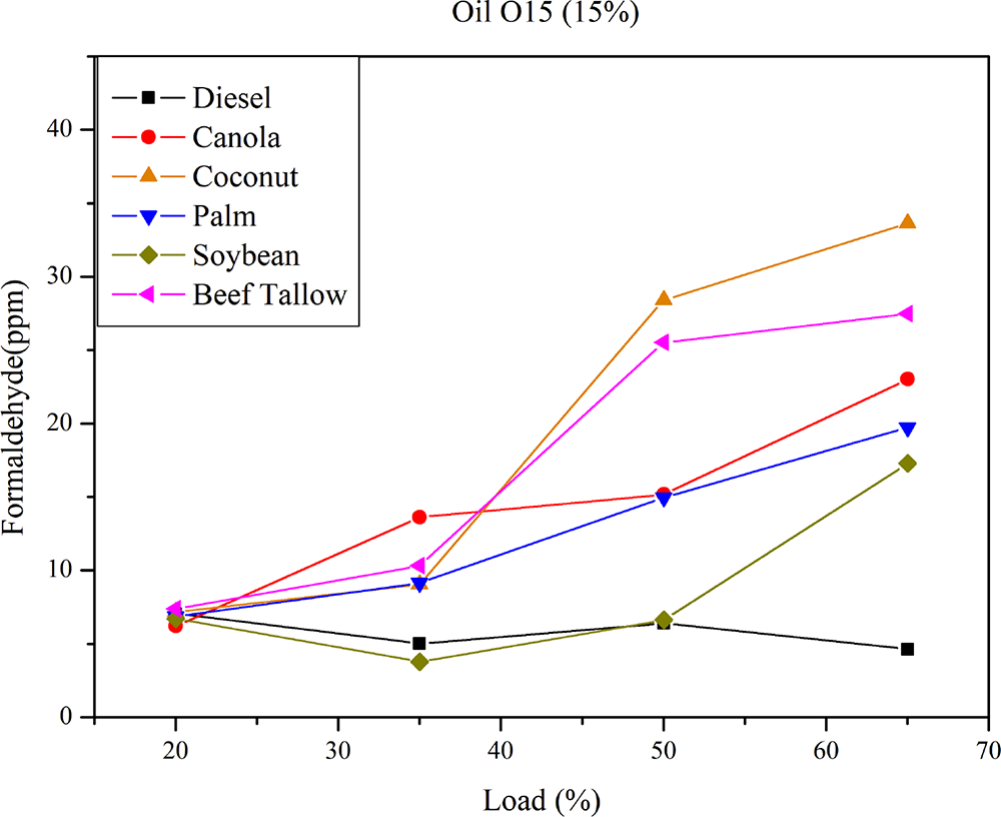
Formaldehyde emissions for diesel–triglyceride mixtures of O15 at different loads.

Among these mixtures, the O15 soybean oil blend exhibits the lowest formaldehyde emissions, comparable to diesel emissions relative to those of other diesel–vegetable oil mixtures, with elevated formaldehyde emissions occurring only at loads exceeding 50%. The data clearly indicate that the oil type influences formaldehyde emissions as load increases. At higher loads (65%), all oils exhibit substantial formaldehyde emissions (greater than 10 ppm), whereas at lower loads (20%), formaldehyde emissions for all oils approximate those of pure diesel. Figure 6 further illustrates the impact of feedstock on formaldehyde production. The highest emission levels are associated with blends containing saturated feedstocks, such as coconut and beef tallow, whereas those based on unsaturated feedstocks exhibit lower emissions. An analysis of variance (ANOVA) identified statistically significant differences in formaldehyde emissions relative to the variables “load” and “iodine value”. Multiple linear regression analysis between formaldehyde emission and both the iodine value and the load yielded a predictive model with an *R* ^2^ value of 0.75, thereby demonstrating the statistical relevance of these variables. The “load” variable produced a positive coefficient of 0.4, indicating that formaldehyde emissions increase with higher load, whereas the “iodine value” presented a negative coefficient of −0.067, signifying a decrease in emissions as iodine index increases. This may be explained by the fact that biofuels with higher iodine values generally possess lower viscosity. Fuels exhibiting high viscosity are less readily atomized within the combustion chamber, forming larger droplets that prolong and complicate the combustion process, thus promoting the formation of unburned hydrocarbons. This phenomenon has also been reported by Hellier et al., who observed a direct relationship between increased unburned hydrocarbon emissions and fuel viscosity, attributing this to impaired atomization.^26^

Figure 7 illustrates formaldehyde emissions for diesel–B15 biodiesel blends under varying load conditions. Formaldehyde emissions consistently exceed those of pure diesel and surpass the levels observed in diesel–triglyceride blends. The biodiesel feedstock material significantly influences formaldehyde emissions as the load increases. Maximum formaldehyde emissions occur at the highest load (65%) for beef tallow, palm, and coconut biodiesel. Minimum formaldehyde emissions (lower than 10 ppm) occur at the lowest load (20%) for soybean and coconut biodiesel.

**Figure 7:**
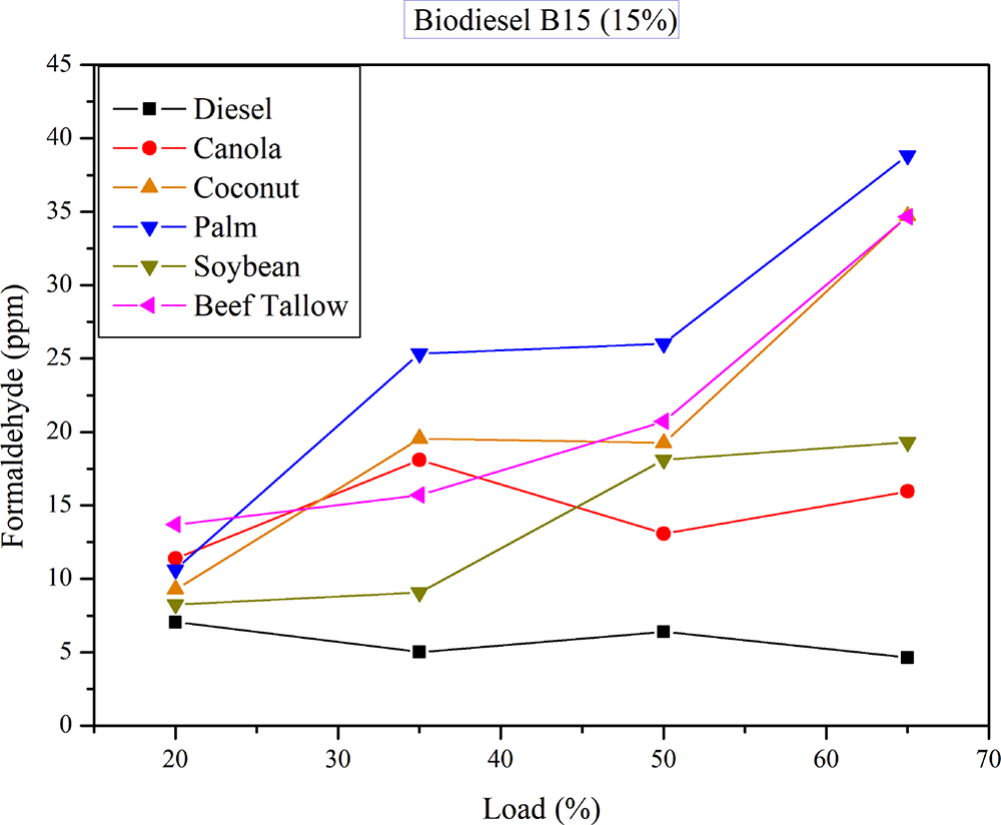
Formaldehyde emissions for B15 diesel–biodiesel mixtures at different loads.

This behavior is similar to that observed for diesel–oil blends. A two-way ANOVA also revealed significant differences in formaldehyde emissions based on both power output and the type of feedstock used. Linear regression analysis demonstrated statistical significance for the variables “load” and “iodine value,” with a correlation coefficient of *R* ^2^ = 0.68, a positive coefficient of 0.37 for load, and a negative coefficient of −0.37 for iodine value. These coefficients indicate that formaldehyde emissions increase with increasing load and decrease with increasing iodine value of the fuel.

Figure 8 presents formaldehyde emissions for the B100 biodiesel. Formaldehyde emissions are significantly elevated in B100 blends compared to diesel. The biodiesel type exerts considerable influence on formaldehyde emissions with increasing load. Maximum formaldehyde emissions occur at the highest load (65%) for canola biodiesel (higher than 35 ppm), followed by soybean biodiesel (≈23 ppm). At 50% load, coconut and beef tallow biodiesel exhibit the highest emission rates, approximately 27 and 24 ppm, respectively. Conversely, minimum formaldehyde emissions occur at the lowest load (20%) for palm, coconut, soybean, and beef tallow biodiesel. Despite the observed trend of higher emissions for saturated feedstocks, under some operating conditions, unsaturated biofuels yield higher formaldehyde emissions. This scenario does not allow to assert a definitive correlation in this direction. ANOVA analysis indicates that there is no statistically significant effect of iodine value on formaldehyde emissions. This result is corroborated by the linear regression analysis, which does not provide strong evidence supporting such a relationship. It is plausible that the impact of feedstock on emissions arises from a combination of properties that do not follow a strictly linear pattern, suggesting the need to incorporate additional variables into the regression models beyond those considered in Table 6.

**Figure 8:**
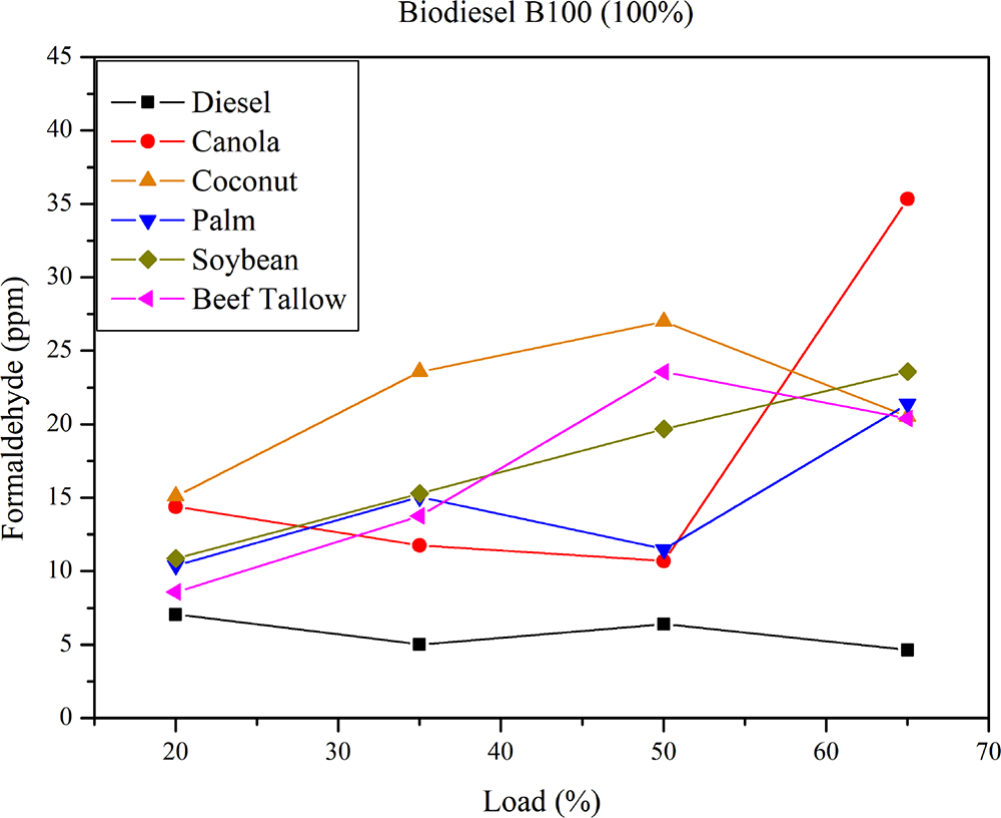
Formaldehyde emissions for diesel and pure biodiesel (B100) at different loads.

The elevated formaldehyde emissions observed with diesel–biodiesel blends compared to pure diesel have been documented in multiple studies.^27–33^ The higher oxygen content typically present in biodiesel contributes to increased carbonyl emissions, as previously reported by Xue et al.^34^ and Liu et al.^27^

#### Acetaldehyde Emission

Figure 9 presents acetaldehyde emissions for diesel–triglyceride mixtures for the O15 complex at varying loads. At 20% load, acetaldehyde emissions from diesel exceed those from O15 mixtures. However, as load increases, acetaldehyde emissions from O15 mixtures surpass those of diesel fuel. The oil type influences acetaldehyde emissions with increasing load. Maximum acetaldehyde emissions occur at the highest load (65%) for beef tallow and coconut oil, while minimum acetaldehyde emissions occur at the lowest load (20%) for palm, canola, and coconut oil. Detected acetaldehyde emissions were found to be lower than those of formaldehyde, rendering statistical analysis less effective in establishing direct relationships between fuel properties and emissions. Nevertheless, a trend similar to that seen in formaldehyde emissions is discernible: feedstocks with lower iodine values tend to produce higher acetaldehyde emissions.

**Figure 9:**
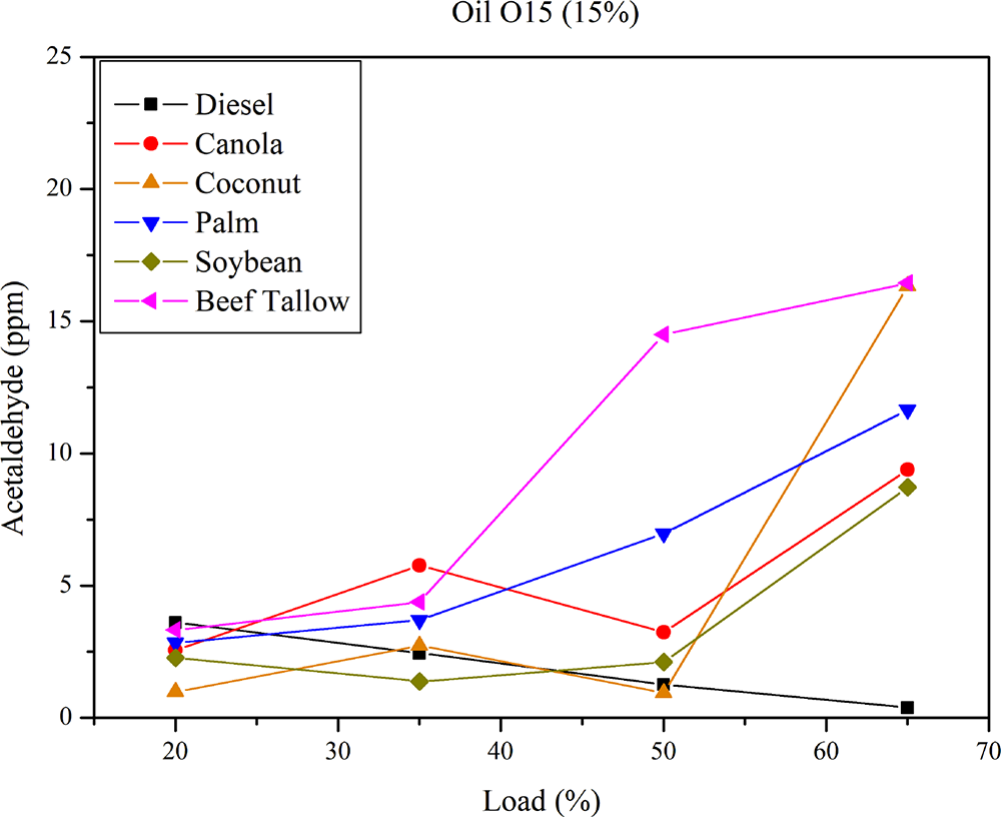
Acetaldehyde emissions for B15 diesel–biodiesel mixtures at different loads.

Figure 10 illustrates the acetaldehyde emissions for B15 diesel–biodiesel mixtures at different loads.

**Figure 10:**
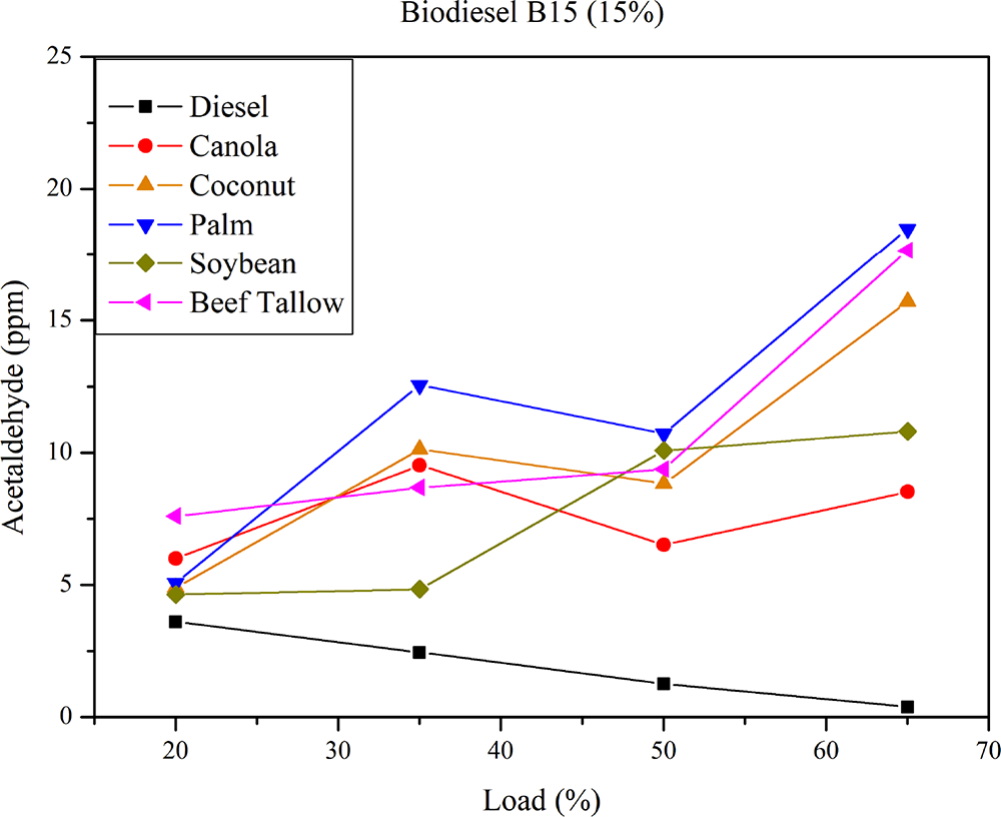
Acetaldehyde emissions for B15 diesel–biodiesel mixtures at different loads.

Acetaldehyde emissions from B15 mixtures consistently exceed those of diesel fuel. The biodiesel type influences acetaldehyde emissions as the load increases. Maximum acetaldehyde emissions occur at the highest load (65%) for palm, beef tallow, and coconut biodiesel, while minimum acetaldehyde emissions occur at the lowest load (20%) for palm, soybean, and coconut biodiesel. As reported for diesel–oil blends, statistical analyses suggest no conclusive correlation between feedstock properties and acetaldehyde emissions. However, examination of regression curves indicates that saturated feedstocks are associated with a greater increase in acetaldehyde emissions as the load increases, whereas unsaturated feedstocks exhibit relatively lower emissions.

Figure 11 presents acetaldehyde emissions for B100 diesel–biodiesel mixtures at varying loads. For all mixtures, acetaldehyde emissions from pure biodiesel (B100) exceed those of pure diesel fuel.

**Figure 11:**
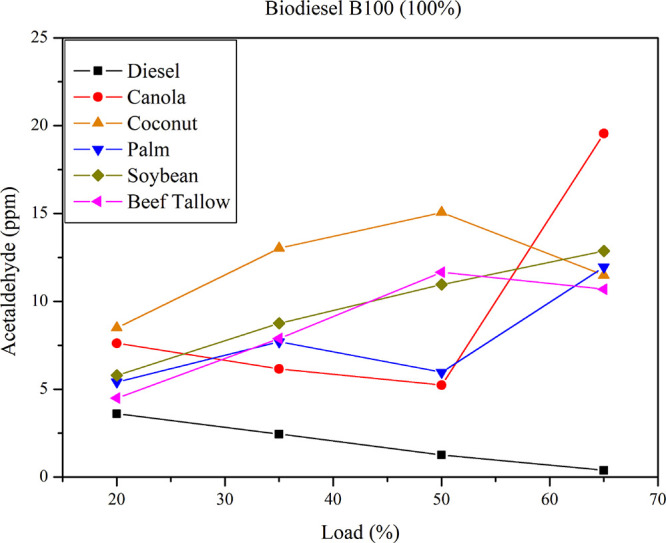
Acetaldehyde emissions for diesel and pure biodiesel (B100) at different loads.

As in previous cases, the biodiesel type influences acetaldehyde emissions with increasing load. Maximum acetaldehyde emissions occur at the highest load (65%) for canola biodiesel and at a 50% load for coconut biodiesel. Soybean biodiesel and beef tallow biodiesel exhibit an almost linear increase in acetaldehyde emissions with increasing load. Minimum acetaldehyde emissions occur at the lowest load (20%) for biodiesel derived from beef tallow, palm oil, and soybean, with emissions approximating those of pure diesel at approximately 5 ppm. The range of acetaldehyde emissions among the tested feedstocks remains narrow, and, based on the conducted statistical and analytical analyses, it is not possible to establish a clear correlation between feedstock type and acetaldehyde emissions.

Previous research has documented variations in acetaldehyde emissions between diesel–biodiesel blends and pure diesel.^27–33,35^ The higher oxygen content typically present in biodiesel contributes to increased carbonyl emissions, as reported by Xue et al.^34^ and Liu et al.^27^ The measured formaldehyde concentrations for both diesel–triglyceride and diesel–biodiesel blends are consistently greater than those of acetaldehyde, indicating a preferential formation of smaller carbonyls.

#### State-of-the-Art Comparison

A review of the literature reveals numerous studies addressing formaldehyde and acetaldehyde emissions from diesel engines, using various blends of diesel and biodiesel. However, there is no clear consensus among the authors on this topic. This lack of agreement can be attributed to the use of biodiesel and diesel fuels with differing origins and ester profiles, as highlighted by Ballesteros et al.^31^ Additionally, operational and sampling conditions vary across the studies, further influencing the results. The quality of biodiesel—characterized by its fatty acid profile, iodine number, and purity—has been shown to affect the formation of specific carbonyl emissions.^35^ The following section provides a detailed discussion of the most recent articles in this field.

Liu et al.^27^ investigated the use of diesel and diesel–palm biodiesel blends (B20, B50, B70, B100) in a four-cylinder, high-pressure, common-rail diesel engine equipped with a turbocharger. Formaldehyde and acetaldehyde emissions were measured using a multicomponent gas analyzer (SESAM-FTIR, AVL, Austria). The study observed that emissions of both formaldehyde and acetaldehyde were higher for diesel–palm biodiesel blends than for pure diesel and decreased as load increased within the 25–100% load range.

Puškár et al.^28^ examined blends of diesel and biodiesel (B25, B50, B75) derived from waste diesel oil in a diesel engine with a common-rail, direct fuel injection system. A multicomponent gas analyzer was used to measure formaldehyde and acetaldehyde emissions. The results indicated that emissions of both compounds increased with a higher proportion of biodiesel in the fuel blend. Furthermore, an increase in formaldehyde emissions was recorded within the interval from a low to a middle engine loading level. A decrease in this kind of emissions was monitored in the range from middle to high engine loading levels and for all the tested fuels, at an engine speed of 1920 rpm. The developed trends concerning acetaldehyde emissions are very similar.

In a separate study, Venu et al.^36^ investigated blends of diesel and Chlorella emersonii biodiesel (B10, B20, B30, B40, B100) in a single-cylinder diesel engine. Their findings demonstrated a decline in formaldehyde and acetaldehyde emissions with increasing engine load from 20 to 100%.

Li et al.^37^ studied a direct-injected, single-cylinder, four-stroke, air-cooled, naturally aspirated diesel engine using diesel and diesel–biodiesel blends (B50, B100). Formaldehyde and acetaldehyde emissions were measured by using solvent extraction and high-performance liquid chromatography (HPLC). The study found that the behavior of formaldehyde and acetaldehyde emissions varied depending on the fuel, sometimes increasing and sometimes decreasing with increasing Brake Mean Effective Pressure (BMPE) within the 0.05–0.5 MPa pressure range.

Man et al.^30^ investigated a four-cylinder, naturally aspirated, direct-injection diesel engine using blends of diesel and waste cooking oil biodiesel (B10, B20, B30, and B100). Emissions of formaldehyde and acetaldehyde were measured with a V&F multicomponent gas analyzer (Ion Molecule Reaction mass spectrometer). Both emissions increased with load from low to medium engine loading at 1920 rpm, but decreased from medium to high engine loading at the same speed. The load range examined was 20–95%.

Tan et al.^38^ studied diesel and diesel–jatropha biodiesel blends (B5, B10, B20, B50, B100) in a light-duty, direct-injection, four-cylinder, four-stroke, turbocharged, intercooled diesel engine with a high-pressure common-rail fuel system. Formaldehyde and acetaldehyde emissions were measured using the AVL SESAM FTIR analyzer. Emissions of both compounds decreased with increasing load in the range of 10–75%.

Di et al.^39^ investigated a four-cylinder, water-cooled diesel engine with direct injection and natural aspiration, at a speed of 1800 rpm, using blends of diesel and biodiesel from used cooking oil (B19.6, B39.4, B59.4, B79.6, B100). An Airsense multicomponent gas analyzer was used to measure emissions. An increase in formaldehyde emissions was recorded in the range between low and medium engine load levels. However, a decrease in acetaldehyde emissions was observed in the range between medium and high engine load levels for all of the fuels tested.

Liu et al.^40^ studied the use of diesel and a diesel–palm biodiesel blend (B10) in a four-cylinder, four-stroke, direct-injection diesel engine. Formaldehyde and acetaldehyde emissions were measured by using high-performance liquid chromatography (HPLC) with an ultraviolet detector. The results showed that emissions of both formaldehyde and acetaldehyde decreased with increasing load in the range of 10–55%.

The synthesis of current literature reveals significant variability in formaldehyde and acetaldehyde emissions from diesel engines fueled with biodiesel–diesel blends, as demonstrated by studies such as Liu et al.,^27^ Puškár et al.,^28^ and Venu et al.^36^ These discrepancies arise from differences in fuel composition (e.g., ester profiles, feedstock origin), engine design (e.g., common-rail vs naturally aspirated systems), and operational parameters (e.g., load, rpm). For instance, Liu et al.^27^ observed decreasing aldehyde emissions with increasing load for palm biodiesel blends, while Puškár et al.^28^ reported the opposite trend for waste-derived biodiesel. Such contradictions underscore the need for a systematic analysis of the factors governing carbonyl formation. As demonstrated in the preceding review, the literature on formaldehyde and acetaldehyde emissions from diesel engines fueled with oxygenated blends remains inconclusive. It is generally observed that oxygenated fuels tend to increase emissions of these aldehydes compared to those of conventional diesel.

## Discussion

The behavior of Specific Fuel Consumption (SFC) as a function of engine load in this study aligns with previously reported findings, which consistently demonstrate a decrease in SFC with increasing load. However, the observed trends in formaldehyde and acetaldehyde emissions deviate from the expected pattern. It is widely accepted that as engine load increases, combustion chamber temperature rises, and emissions of unburned hydrocarbons typically decrease. This general trend is evident in Figures 6–11 for diesel fuel. In contrast, the biofuel blends evaluated in this investigation exhibit an inverse behavior, wherein formaldehyde and acetaldehyde emissions increase with increasing load.

This counterintuitive phenomenon can be attributed to the distinct chemical and physical properties of the oxygenated biofuels studied. These fuels possess a high oxygen content within their molecular structure, longer hydrocarbon chains, greater viscosity, elevated ignition temperatures, and lower calorific values. Mechanistically, these properties contribute to fuel atomization challenges, resulting in the formation of larger droplets that remain within the combustion chamber. Such droplet behavior leads to delayed combustion and consequently reduces the peak combustion temperature.^26^

Moreover, the elevated oxygen content in the fuel enhances the concentration of oxygen radicals during combustion. When combustion occurs late, these radicals facilitate the formation of intermediate oxidation products, predominantly formaldehyde and acetaldehyde.^41^ The combined effect of incomplete atomization, lower temperature profiles, and fuel-derived oxygen radicals results in increased aldehyde emissions despite the overall decrease in SFC. This interplay explains the observed divergence from conventional expectations, highlighting the complex combustion dynamics associated with oxygenated biofuels.

It is important to note that within the context of the combustion process of large molecules, an increase in pressure invariably leads to an increase in temperature. This, in turn, initiates a process of cracking the fuel molecules into smaller molecules, including methane and ethane.^42^ These smaller molecules initiate the reaction kinetics, where the most probable outcome is their oxidation and the subsequent formation of CO_2_. However, the reactions of these molecules may involve their oxidation and dehydrogenation in the combustion chamber to form aldehydes.^43^ Given the higher methane concentration, the most likely molecule to form from this species is formaldehyde, also due to its lower enthalpy of formation compared to acetaldehyde. In consideration of the proportionality of the appearance of formaldehyde and acetaldehyde, it is observed that this concentration is a response to a series of simultaneous phenomena. These include the kinetic constant of formaldehyde formation being greater than that of acetaldehyde, the concentration of methane in the chamber being greater than that of ethane as a product of the reaction kinetics of larger molecules, and the energy of formaldehyde formation being less than that of acetaldehyde.^3,42^

However, the question that must be posed is why the emission increases with the load, given that it has been reported for most articles that this concentration decreases.^44^ This is based on the fact that increasing the load increases the temperature in the chamber, causing greater combustion kinetics and favoring the final burning of the aldehydes until they are converted into CO_2_. In this instance, it is hypothesized that an increase in the load results in an augmentation of the fuel injection into the same chamber, thereby engendering a heightened concentration of fuel in relation to the air available for combustion (i.e., a rich mixture).^37^ Conversely, given that the tests in this study are conducted at the same RPM, it is reasonable to assume that the time available for combustion will remain constant. This suggests that a higher proportion of molecules may not achieve complete combustion, resulting in the formation of intermediate products, such as aldehydes. This is particularly evident in the case of oil and biodiesel, whose properties make it difficult to spray them into the camera, resulting in larger droplets remaining in the atmosphere. This, in turn, slows the combustion process, thereby explaining why these biofuels exhibit higher aldehyde emissions than diesel. Furthermore, the presence of oxygen in biofuels during the cracking process has been shown to promote the direct formation of aldehydes.^37,45^ It is evident that the processes involved in the combustion of biodiesels are of a highly complex nature. It is therefore recommended that further studies be conducted in an attempt to elucidate these problems.

## Conclusions

The present study analyzes the emission rates of formaldehyde and acetaldehyde in the exhaust of a diesel engine operating on a mixture of triglycerides and biodiesel in diesel, as a function of load. The evaluation of these emissions was conducted utilizing an optical method, namely, differential optical absorption spectroscopy (DOAS), which has been demonstrated to be remarkably efficacious in the context of such studies. It has been demonstrated that the incorporation of triglycerides and biodiesel into diesel engines results in an augmentation of formaldehyde and acetaldehyde emissions. The specific type of triglyceride and biodiesel employed exerts a discernible influence on the emission levels of these compounds, with the magnitude of the influence correlating with the increase in the load. It has been established that, in general, formaldehyde and acetaldehyde emissions are higher at high loads than at low loads, probably as a response to a rich fuel/air ratio in the engine and an incomplete combustion process.

## Abbreviation

SO_2_: sulfur dioxide
CO: carbon monoxide
NO_*x*_: nitrogen oxides
CH_2_O: formaldehyde
CH_3_CHO: acetaldehyde
HC: hydrocarbonates
FAME: fatty acid methyl esters
DOAS: differential optical absorption spectroscopy
SFC: specific fuel consumption
